# Implementation of full/half bowtie filter models in a commercial treatment planning system for kilovoltage cone‐beam CT dose estimations

**DOI:** 10.1120/jacmp.v17i2.5988

**Published:** 2016-03-08

**Authors:** Sangroh Kim, Parham Alaei

**Affiliations:** ^1^ Department of Radiation Oncology Baylor Scott and White Health Temple TX USA; ^2^ Department of Radiation Oncology University of Minnesota Minneapolis MN USA

**Keywords:** bowtie filter, CBCT, collapsed‐cone convolution, Monte Carlo, CT dose index

## Abstract

The purpose of this study was to implement full/half bowtie filter models in a commercial treatment planning system (TPS) to calculate kilovoltage (kV) cone‐beam CT (CBCT) doses of Varian On‐Board Imager (OBI) kV X‐ray imaging system. The full/half bowtie filter models were created as compensators in Pinnacle TPS using MATLAB software. The physical profiles of both bowtie filters were imported and hard‐coded in the MATLAB system. Pinnacle scripts were written to import bowtie filter models into Pinnacle treatment plans. Bowtie filter‐free kV X‐ray beam models were commissioned and the bowtie filter models were validated by analyzing the lateral and percent‐depth‐dose (PDD) profiles of anterior/posterior X‐ray beams in water phantoms. A CT dose index (CTDI) phantom was employed to calculate CTDI and weighted CTDI values for pelvis and pelvis‐spotlight CBCT protocols. A five‐year‐old pediatric anthropomorphic phantom was utilized to evaluate absorbed and effective doses (ED) for standard and low‐dose head CBCT protocols. The CBCT dose calculation results were compared to ion chamber (IC) and Monte Carlo (MC) data for the CTDI phantom and MOSFET and MC results for the pediatric phantom, respectively. The differences of lateral and PDD profiles between TPS calculations and IC measurements were within 6%. The CTDI and weighted CTDI values of the TPS were respectively within 0.25 cGy and 0.08 cGy compared to IC measurements. The absorbed doses ranged from 0 to 7.22 cGy for the standard dose CBCT and 0 to 1.56 cGy for the low‐dose CBCT. The ED values were found to be 36‐38 mSv and 7‐8 mSv for the standard and low‐dose CBCT protocols, respectively. This study demonstrated that the established full/half bowtie filter beam models can produce reasonable dose calculation results. Further study is to be performed to evaluate the models in clinical situations.

PACS number(s): 87.57.uq

## I. INTRODUCTION

With the advances in cone‐beam computed tomography (CBCT) technology, image‐guided radiation therapy (IGRT) has become the routine method to accurately deliver the therapeutic radiation dose to the target while minimizing unnecessary irradiation to normal tissues. By performing the CBCT imaging prior to radiation treatment, the treatment setup can be verified and fine‐tuned while the patient is on the treatment table. Despite these great benefits, there is concern about the CBCT dose if used frequently during the course of treatment. Therefore, the accurate estimation of the CBCT dose is essential to predict any potential detrimental effects.

There have been many studies performed to estimate the CBCT doses. Islam et al.^(1)^ performed ion chamber and metal‐oxide semiconductor field‐effect transistor (MOSFET) measurements in head and body phantoms for the X‐ray Volume Imaging (XVI) system (Elekta Oncology Systems, Norcross, GA). The maximum dose for the body phantom was found in the range from 1.8 to 2.3 cGy for 120 kVp and from 2.8 to 3.5 cGy for 140 kVp beams. For the head phantom, the maximum dose values were from 1.5 to 2.0 cGy for 100 kVp and from 2.6 to 3.4 cGy for 120 kVp beams. Kan et al.^(2)^ measured organ and effective dose (ED) measurements for the On‐Board Imager (OBI) system (Varian Medical Systems, Palo Alto, CA) by using thermoluminescent dosimeters (TLD) in three different sites. They found that the mean skin doses from standard dose CBCT to head and neck, chest, and pelvis were 6.7, 6.4, and 5.4 cGy per scan, respectively. The ED from standard dose CBCT to head and neck, chest, and pelvis were 10.3, 23.7, and 22.7 mSv per scan, respectively. Song et al.^(3)^ estimated the weighted computed tomography dose indices (${\text{CTDI}}_{W}$) of the CBCT for both XVI and OBI systems. In their study, the ${\text{CTDI}}_{W}$ for OBI were found to be 8.3 cGy for a head scan and 5.4 cGy for a body scan in standard dose mode. Although these previous studies provided the level of the CBCT doses for various CBCT systems, all their data were acquired in the phantom geometries, which quite differ from the anatomies of patients.

Alaei et al.^4^, ^5^, ^6^ explored the possible use of a commercial treatment planning system (TPS) to estimate the dose in the kilovoltage range, including that of kV CBCT. They generated kilovoltage X‐ray energy deposition kernels using Electron Gamma Shower (EGS) 4 SCASPH user code^(7)^ and implemented them in the Pinnacle TPS (Philips Medical Systems, Andover, MA; formerly ADAC Laboratories, Milpitas, CA) in 1999.^(4)^ They subsequently performed dose calculations for a 120 kV static X‐ray beam by using Pinnacle TPS in 2000.^(5)^ They found that the TPS calculations were overall within 2% for the materials of atomic number less than and equal to that of water compared to the TLD measurements. However, it was also found that the TPS calculations did not produce accurate dose values in and around bone material. They suggested the modification of CT number‐to‐density conversion table in the Pinnacle TPS as a remedy for the inaccuracy. After these primary investigations, they extended their work further to the dose estimation of the CBCT imaging for Varian OBI using the Pinnacle TPS in 2010.^(6)^ They found that the TPS calculated doses agreed with TLD measurements in the range of 0% to 19% in soft tissue. Near and within the bone, larger variations were observed. In this later study, they utilized the wedge function to model the X‐ray beam attenuation effect of the bowtie filter in the Pinnacle TPS. Note that, due to the restriction of the Pinnacle wedge function, they were able to implement only the half‐bowtie filter model, and not the full bowtie one. The wedge function in the Pinnacle TPS only accepts monotonically decreasing wedge profiles, which makes the implementation of the full‐bowtie filter model infeasible.

To overcome the limitation of the wedge function, our current study employed a compensator function to implement both full and half‐bowtie filter models in Pinnacle. We focused on the flexible feature of the compensator model, which allows building the concave shape of the full bowtie filter. After implementing the full and half‐bowtie filter compensator models, we generated and commissioned the kV X‐ray beam models and also validated the bowtie filter models by comparing the calculation results with ion chamber (IC) measurements and Monte Carlo (MC) simulations. We also performed CBCT dose calculations using a CT dose index (CTDI) body phantom and compared the calculation results with IC and MC data. To explore the application of the established models further, we investigated absorbed doses and ED calculations for the CBCT protocols with a five‐year‐old pediatric anthropomorphic phantom and the results were compared with MOSFET measurements and MC data. It should be mentioned that this is the first study which implemented/commissioned the full‐bowtie filter model in any treatment planning system and applied it to the CBCT dose calculations completely. We expect that this study provides valuable information on CBCT dose estimations to patients.

## II. MATERIALS AND METHODS

This study was performed by following these steps: 1) addition of the low energy deposition kernels to the Pinnacle TPS, 2) generation of full and half bowtie compensator models, 3) commissioning/validation of the full and half bowtie X‐ray beam models, 4) calculation of CBCT imaging doses in the CTDI phantom and CTDI/weighted CTDI estimations, and 5) calculation of CBCT doses in the anthropomorphic phantom and absorbed dose and ED estimations. The process of each step will be described in detail.

The low‐energy deposition kernels created by Alaei et al.^(4)^ were added to a retired Pinnacle TPS (version 9.6, Philips Healthcare, Best, Netherlands) which is currently not used for patient treatments. Detailed information about the kernels’ generation can be found in the publication of Alaei et al.^(4)^

The full‐ and half‐bowtie filter beam models of a Varian OBI system were developed in the Pinnacle TPS. Both bowtie filter beam models were created as compensators in the Pinnacle using MATLAB software (Version 2011a, MathWorks, Natick, MA). First, the physical profiles and dimensions of both bowtie filters were acquired from the manufacturer under a nondisclosure agreement, imported into the MATLAB system, and hard‐coded into binary file format. Due to a restriction within Pinnacle, the compensator model must be located at the source‐surface distance (SSD) greater than 30 cm, so the profiles of the compensator models were interpolated by considering the geometric magnification effect. The compensators were set to be placed at the SSD=33 cm in the Pinnacle beam modifier section, which is different than the actual location of the bowtie filter (10 cm away from the source). Second, a Pinnacle script was written to load each bowtie filter compensator data into a Pinnacle treatment plan as a compensator. All the parameters for the compensators, such as physical density, location, output factor, and resolution, were stored in the script and loaded into the treatment plan when the script was executed.

In the Pinnacle physics module, two kV X‐ray beam models, without the bowtie filters, were created and commissioned based on the lateral and percent depth‐dose (PDD) profile data obtained from MC simulations, which were benchmarked with IC measurements in a previous study.^(8)^ Note that each kV X‐ray beam model contains different blade geometry setting that corresponds to each bowtie CBCT scanning protocol (i.e., full bowtie imaging mode uses a symmetric beam aperture, while the half bowtie imaging mode uses an off‐axially shifted beam aperture). The detailed X‐ray beam parameters and geometry settings are presented in Table 1. The commissioning process was similar to the previous study of Alaei et al.^(6)^ The X‐ray beam spectrum data were iteratively adjusted to match the Pinnacle‐calculated lateral and PDD profile data to those of the MC simulations.

After the commissioning of the two bowtie filter‐free kV X‐ray beam models, the validation of the bowtie filter compensator models was performed. A cuboid water phantom ($50\times 50\times 50\;{\text{cm}}^{3}$) was generated in the TPS and an anterior/posterior static 125 kVp X‐ray beam was placed on the surface of the phantom at SSD=100 cm. For each beam model, corresponding bowtie filter compensator was loaded to the corresponding treatment plan by executing the Pinnacle script. Dose calculation for each bowtie filter was performed using collapsed cone convolution (CCC) dose calculation algorithm.^(9)^ Lateral dose profile at the depth of 3 cm and PDD data of the IC measurements and MC simulations from the previous study^(8)^ were used to compare with the data extracted from the Pinnacle treatment plans. The location and physical density of the bowtie filter were iteratively adjusted to achieve the best match in the lateral profiles and PDD data. Note that the treatment table was not included in the dose calculations because the cuboid phantom was large enough to compensate the backscattering effect of the treatment table.

**Table 1:** X‐ray beam parameters and geometry settings for full/half fan CBCT scan geometries of Varian OBI

	*CTDI Body Phantom*		*5‐Year‐Old Pediatric Phantom*	
	*Pelvis‐Spotlight*	*Pelvis*	*Standard Dose Head*	*Low Dose Head*
Peak Voltage (kVp)	125	125	125	125
Tube Current (mA)	80	80	80	40
Exposure Time (ms)	25	13	25	10
Rotation Range (deg)	200	360	360	360
Number of Projection	376	679	650‐700	650‐700
Fan Type	Full Fan	Half Fan	Full Fan	Full Fan
Blade Setting, X	13.6 / 13.6	6.8 / 23.5	13.6 / 13.6	13.6 / 13.6
Blade Setting, Y	10.3 / 10.3	10.3 / 10.3	10.3 / 10.3	10.3 / 10.3
Slice Thickness (mm)	2.5	2.5	2.5	2.5

The beam outputs of the bowtie filter‐free kV X‐ray beam models were initially measured and computed according to the AAPM TG‐61 protocol^(10)^ with a parallel plate ion chamber (Model: A600, Standard Imaging, Middleton, WI) in a Virtual Water phantom (Standard Imaging, Middleton, WI) and were entered in the bowtie beam models in Pinnacle TPS as cGy/MU with 1 MU being equivalent to 1 min, following the previous study of Alaei et al.^(6)^ To compensate the limitation of kV beam output measurements in Virtual Water,^(11)^ the beam output was fine‐tuned to produce the best match with the IC measurements and MC data from the previous study of Kim et al.^(8)^

**Figure 1:**
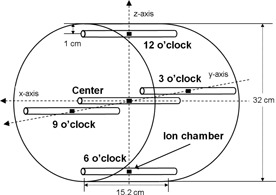
Experimental setup of IC measurements in a CTDI body phantom. Note that an IC is separately placed in the middle of five locations (center and four peripheries) per each CBCT irradiation.

After the validation of the bowtie filter models, CTDI calculations for two CBCT imaging protocols were performed using the CTDI body phantom in Pinnacle. The CTDI phantom was introduced for the purpose of calculating the CBCT doses in a simple cylindrical geometry as initial tests. The CTDI body phantom is made of polymethylmethacrylate (PMMA) with density $\rho =1.19\;g/{\text{cm}}^{3}$ with the diameter of 32 cm and the length of 15.2 cm. As shown in Fig. 1, five measurement locations were selected (one central and four peripheral) within the CTDI phantom. The locations of the four peripheral dose calculations were 1 cm below the surface of the CTDI phantom. It should be mentioned that this study assumed the point doses are equivalent with CTDI values in the CBCT geometry following Dixon's study.^(12)^ Pelvis spotlight and pelvis CBCT imaging protocols of Varian OBI (version 1.4) were employed in the dose calculations of the Pinnacle TPS. The detailed CBCT scan parameters are presented in Table 1. The calculated doses were compared with the IC measurements and MC simulations described in a previous study.^(13)^ In addition, weighted CTDI values^(14)^ were calculated using Eq. (1):

(1) $$CTDIw={\;}^{1}\;/\;\;{\;}_{3}CTDI_{center}+{\;}^{2}\;/\;\;{\;}_{3}CTDI_{peripheries},$$

where ${\text{CTDI}}_{\text{center}}=\text{a point dose at center}$, and ${\text{CTDI}}_{\text{peripheries}}=\text{an average dose at peripheries}$.

For the application of CBCT dose calculations in a more realistic geometry, a five‐year‐old pediatric anthropomorphic phantom (Model ATOM 705‐D, CIRS, Norfolk, VA) was employed and the absorbed doses from standard dose head and low‐dose head CBCT protocols (Varian OBI version 1.3) were calculated in Pinnacle TPS. The calculated data were compared with MOSFET measurements and MC simulation data obtained from previous work.^(15)^ Note that the CBCT protocols of Varian OBI version 1.3 were used in the Pinnacle TPS calculation to match the version of the CBCT protocols used in the MOSFET measurements and MC simulations. This study assumed that the absorbed dose of individual organ is equivalent with the point dose measured/calculated at the corresponding organ location. With the absorbed dose data, ED values were also estimated by applying the tissue weighting factors from ICRP publication 60^(16)^ to the equivalent doses, which were the same as organ doses used for photon irradiation:

(2) $$ED=\sum W_{ri}H_{i},$$

where $W_{\text{ri}}=\text{tissue weighting}$ factor of individual organ *i*, and $H_{i}=\text{equivalent}$ dose of individual organ *i*.

The detailed calculation method of organ and ED values can be found in the previous study.^(15)^

Note that the treatment table was not included in both CTDI and CBCT dose calculations.

## III. RESULTS

**Figure 2:**
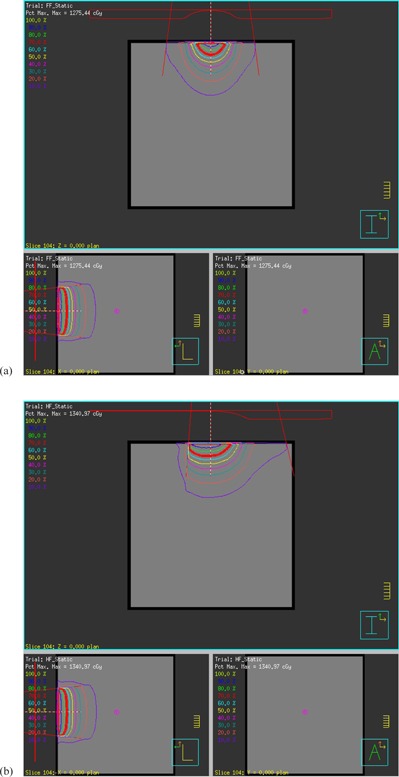
Pinnacle dose distributions of a kV static X‐ray beam with (a) full‐bowtie filter geometry and (b) half‐bowtie filter geometry in the cuboid water phantom.

**Figure 3:**
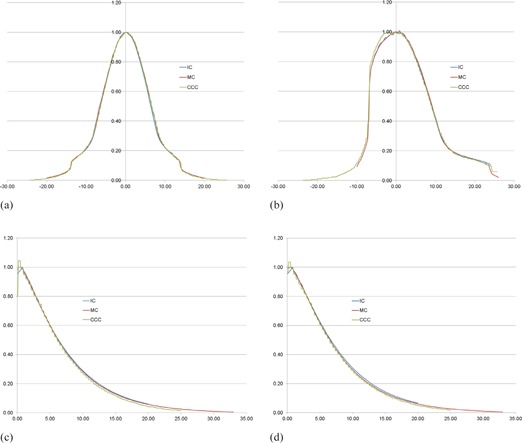
Comparison of lateral and PDD profiles among Pinnacle dose calculations, IC measurements, and MC simulations: (a) lateral profiles of full‐bowtie filter model, (b) lateral profiles of half‐bowtie filter model, (c) PDD profiles of full‐bowtie filter model, and (d) PDD profiles of half‐bowtie filter model. The x‐axis is in cm.

Figures 2(a) and 2(b) show the Pinnacle dose distribution of a kV static X‐ray beam with full‐and half‐bowtie filter geometries in the cuboid water phantom acquired during the validation process of the bowtie filter models. As aforementioned, the kV beam models were commissioned by comparing the lateral and PDD profiles of the calculation results with the IC measurements and MC simulations brought from the previous study of Kim et al.^(8)^ Comparison of the results of the three methods is presented in Figs. 3(a) to 3(d). As can be seen, most of the profiles were well matched except for a few regions that will be discussed further. It was also found that the differences of lateral and PDD profiles among the three methods were within 6%.

**Figure 4:**
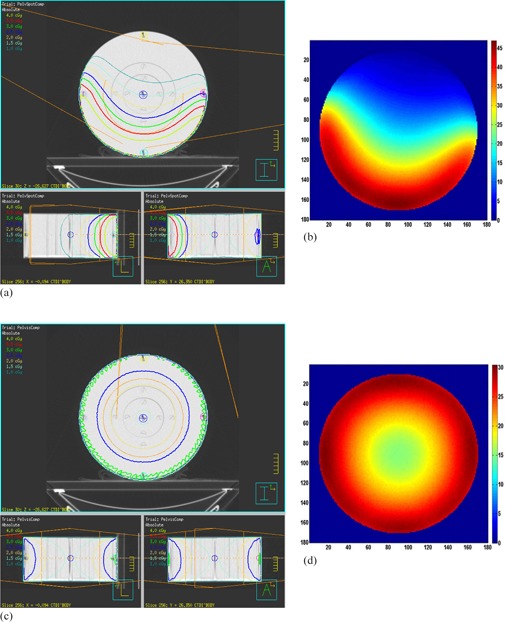
Dose distributions of kV CBCT imaging protocols in the CTDI body phantom: pelvis spotlight CBCT protocol with full‐bowtie filter in (a) Pinnacle TPS and (b) MC simulations, and pelvis CBCT protocol with half‐bowtie filter in (c) Pinnacle TPS and (d) MC simulations. *MC data is presented with permission of published journal.^(13)^ Note that the color scale in the MC simulations is in mGy.

Figures 4(a) to (d) show the dose distributions of the kV CBCT protocols in the CTDI body phantom. Figures 4(a) and 4(b) show pelvis spotlight CBCT protocol with full‐bowtie filter in the Pinnacle TPS and MC simulations, and Figs. 4(c) and 4(d) show pelvis CBCT protocol with half‐bowtie filter in the Pinnacle TPS and MC simulations. As seen in the figures, the dose distributions from Pinnacle's CCC dose calculations and MC simulations are quite similar to one another. Note that the dose distributions of the two CBCT protocols are different due to the different rotation ranges— 200° half fan in pelvis spotlight and 360° full fan in pelvis protocols. The CTDI and ${\text{CTDI}}_{W}$ values from Pinnacle TPS, IC measurements and MC simulations in the CTDI body phantom are presented in Table 2. It was found that all the CTDI and ${\text{CTDI}}_{W}$ values of the three methods were relatively close; dose differences between IC measurement and Pinnacle's CCC dose calculations were within 0.03 cGy and 0.08 cGy for pelvis spotlight and pelvis CBCT protocols, respectively.

**Figure 5:**
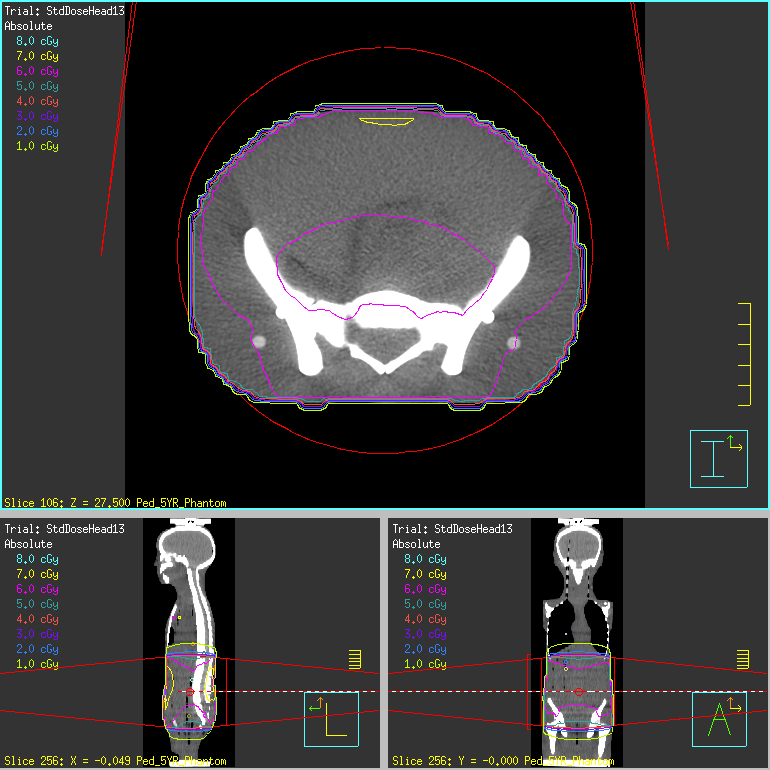
Pinnacle dose distributions of a kV standard dose CBCT imaging protocol in five‐year‐old pediatric anthropomorphic phantom. Note that the CBCT imaging is calculated for the abdominal irradiation.

**Figure 6:**
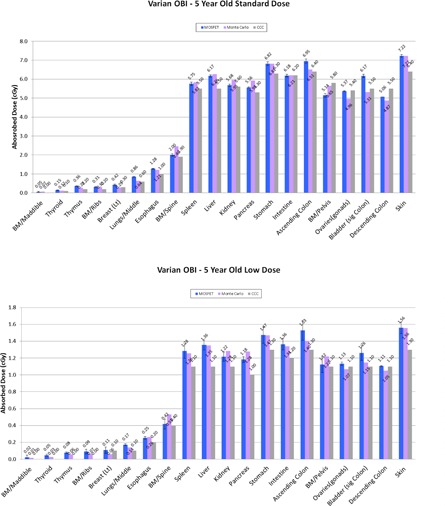
Comparison of absorbed dose distributions among MOSFET measurements, MC data, and Pinnacle calculations from (a) standard dose head CBCT imaging protocol and (b) low‐dose head CBCT imaging protocol. Note that the absorbed dose is assumed to be equivalent with the measured/calculated point dose. *MOSFET and MC data are presented with permission of published journal.^(15)^ BM=bone marrow; CCC=Pinnacle collapsed cone convolution.

Figure 5 presents the Pinnacle dose distributions calculated from a kV standard dose CBCT protocol in the 5‐year‐old pediatric anthropomorphic phantom. Note that the CBCT imaging doses were calculated for the abdominal irradiation. Figures 6(a) and 6(b) show the results of the absorbed dose calculations among the Pinnacle's CCC dose calculations, MOSFET measurements, and MC simulations. It was found that the absorbed doses of the three methods were ranged from 0 to 7.22 cGy for the standard dose CBCT protocol and from 0 to 1.56 cGy for the low‐dose CBCT protocol. As seen in the figures, all three methods show reasonable agreement. The ED values for the standard dose CBCT protocol were found to be 37.8, 36.1, and 35.9 mSv for MOSFET, MC, and Pinnacle TPS, respectively. Those for the low‐dose CBCT protocol were 8.1, 7.8, and 7.2 mSv for MOSFET, MC, and Pinnacle TPS, respectively.

**Table 2:** Comparison of CTDI and weighted CTDI values among TPS dose calculations, ion chamber measurements and MC simulations

	*Pelvis Spotlight*					*Pelvis*				
	*IC* ^a^	*CCC*	*MC* ^a^	*IC‐CCC*		*IC* ^a^	*CCC*	*MC* ^a^	*IC‐CCC*	
*Location* ^b^	*Dose (cGy)*	*Dose (cGy)*	*Dose (cGy)*	*Diff. (cGy)*	*Diff.%*	*Dose (cGy)*	*Dose (cGy)*	*Dose (cGy)*	*Diff. (cGy)*	*Diff. (%)*
Center	1.65	1.50	1.50	0.15	9.1	1.75	1.50	1.54	0.25	14.3
12 O'clock	0.26	0.20	0.14	0.06	23.1	3.05	2.90	3.02	0.05	1.6
3 O'clock	2.43	2.40	2.24	0.03	1.2	2.96	3.00	3.01	−0.04	−1.4
6 O'clock	4.42	4.60	4.67	−0.19	−4.3	2.87	3.00	2.93	−0.13	−4.5
9 O'clock	3.86	3.90	4.07	−0.04	−1.0	2.89	2.90	3.01	−0.01	−0.3
${\text{CTDI}}_{W}$	2.38	2.35	2.35	0.03	1.3	2.54	2.47	2.51	0.08	3.1

a ^a^ IC and MC data are presented with permission of published journal.^(13)^

b ^b^ Location refers to that in Fig. 1.

IC=ion chamber; CCC=collapsed cone convolution; MC=Monte Carlo.

## IV. DISCUSSION

We implemented the full/half bowtie filter models of the Varian OBI system in the Pinnacle TPS to enable the kV CBCT imaging dose assessments. As the initial geometric verification step, the shapes and orientations of the bowtie filter models were visually inspected as well as the dose distributions of the kV static X‐ray beams with full/half bowtie filters in the water phantom (see Fig. 2); these visual examinations qualitatively verified the geometrical accuracies of the bowtie filter model implementations.

For the second verification step, the dosimetric properties of the static X‐ray beams with the bowtie filter models were investigated; the lateral and PDD profiles of the bowtie filter models were compared with IC measurements and MC simulations. As seen in Fig. 3, the lateral and PDD profiles in the bowtie filter model validation process were well matched to each other except for a few regions: 1) the lateral profiles on the x‐axis coordinates from ‐7 to 0 in Fig. 3(b), and 2) the PDD profiles at the surface area of the cuboid phantoms in Figs. 3(c) and 3(d). The lateral profile discrepancies among the three methods in Fig. 3(b) were caused by the beam hardening effect of the bremsstrahlung X‐rays in the OBI X‐ray tube which the CCC dose calculation model in the Pinnacle TPS cannot handle appropriately. Note that photon beam model of the Pinnacle CCC dose calculation engine accepts only a single polyenergetic photon beam energy spectrum, which does not consider the variations of the energy spectra at off‐axial locations from the central axis of the real X‐ray target induced by the beam hardening effect.^(17)^ This effect was not seen in the MC data because the simulations of bremsstrahlung X‐ray generations were included in the MC calculations that account for the beam hardening effect inside the simulations. The dose discrepancies of the PDD profiles at the interface between the air and the surface of the water phantom were understandable phenomena. It is well known that CCC algorithm shows limited calculation accuracies at the interface of two inhomogeneous materials, compared to the MC method.^(18)^ It should also be noted that IC measurements showed large differences compared with the MC data, which are caused by the lack of the charged particle equilibrium and volume averaging effect.

To verify the bowtie filter models in the cylindrical geometry, the CTDI body phantom was introduced and the CTDI and ${\text{CTDI}}_{W}$ were calculated and compared with IC measurements and MC results. It was found that there was no significant difference in CTDI and ${\text{CTDI}}_{W}$ values among the three methods presented in Table 2. Notice that there is no treatment table included in the CCC and MC calculations. Also, as can be seen in Fig. 4, the two‐dimensional dose distributions between the Pinnacle TPS calculations and MC simulations were found to be qualitatively close to each other. From these results, we confirmed that the implemented bowtie filter models were found to be applicable to the CBCT dose estimations in the cylindrical geometry.

To extend the application of the bowtie filter models, we employed the five‐year‐old pediatric anthropometric phantom to calculate the absorbed doses and ED from the CBCT imaging protocols. It was found that the maximum differences of the absorbed doses between MOSFET measurements and the Pinnacle's CCC dose calculations were 0.8 cGy and 0.3 cGy for the standard dose head and low‐dose head CBCT protocols, respectively. Additionally, the ED values between MOSFET measurements and the Pinnacle's CCC dose calculation were found to be within 5% and 11%. These results demonstrated that the full/half bowtie filter models in the Pinnacle TPS can produce reasonably accurate absorbed dose and ED values in the anthropomorphic phantom geometry.

There is some extensive work to be done in the future. 1) This study only investigated two CBCT imaging protocols that are frequently used in the clinic. The kV beam models for other CBCT imaging protocols will be investigated in the future. 2) The bowtie filter models were only implemented for the certain models of Varian's OBI system (Varian Clinac EX, IX, Trilogy series) To be applicable to the TrueBeam kV imaging system, new full/half bowtie filter models for the TrueBeam kV X‐ray beam need to be established and commissioned, which is beyond the scope of this study and will be explored in future work. 3) The implemented bowtie filter models were applied only to the cylindrical and anthropomorphic phantoms and not to the real patient body habitus. Again, further study will be performed in the future.

## V. CONCLUSIONS

In this study, the full/half bowtie filter kV X‐ray beam models for CBCT imaging protocols were established and commissioned in the Pinnacle TPS. With the CTDI and five‐year‐old pediatric anthropomorphic phantoms, both bowtie models were also validated by comparing the calculation results with the IC, MOSFET measurements, and MC simulations. It was found that the established kV X‐ray beam models can provide reasonable accuracies for the CBCT dose calculations in water, CTDI, and five‐year‐old pediatric anthropomorphic phantoms. Implemented bowtie filter models can be applicable for the CBCT imaging dose assessments in radiation therapy patients.

## ACKNOWLEDGMENTS

We thank Dr. Jeffrey Siebers of University of Virginia for discussion regarding Pinnacle scripting, Dr. Terry Yoshizumi of Duke University for the permission of ion chamber and MOSFET data usage, and Mr. Todd Racine of Advocate Condell Medical Center for the access of Pinnacle TPS system. This study was presented as a snap‐oral presentation at the AAPM 2015 annual meeting, Anaheim, CA.

## COPYRIGHT

This work is licensed under a Creative Commons Attribution 4.0 International License.
